# Linear models for diallel crosses: a review with R functions

**DOI:** 10.1007/s00122-020-03716-8

**Published:** 2020-11-06

**Authors:** Andrea Onofri, Niccolò Terzaroli, Luigi Russi

**Affiliations:** Dipartimento di Scienze Agrarie, Alimentari e Ambientali, Universita’ Degli Studi di Perugia, Borgo XX Giugno, 74, 06121 Perugia, Italy

## Abstract

**Key message:**

A new R-software procedure for fixed/random Diallel models was developed. We eased the diallel schemes approach by considering them as specific cases with different parameterisations of a general linear model.

**Abstract:**

Diallel experiments are based on a set of possible crosses between some homozygous (inbred) lines. For these experiments, six main diallel models are available in literature, to quantify genetic effects, such as general combining ability (GCA), specific combining ability (SCA), reciprocal (maternal) effects and heterosis. Those models tend to be presented as separate entities, to be fitted by using specialised software. In this manuscript, we reinforce the idea that diallel models should be better regarded as specific cases (different parameterisations) of a general linear model and might be fitted with general purpose software facilities, as used for all other types of linear models. We start from the estimation of fixed genetical effects within the R environment and try to bridge the gap between diallel models, linear models and ordinary least squares estimation (OLS). First, we review the main diallel models in literature. Second, we build a set of tools to enable geneticists, plant/animal breeders and students to fit diallel models by using the most widely known R functions for OLS fitting, i.e. the ‘lm()’ function and related methods. Here, we give three examples to show how diallel models can be built by using the typical process of GLMs and fitted, inspected and processed as all other types of linear models in R. Finally, we give a fourth example to show how our tools can be also used to fit random/mixed effect diallel models in the Bayesian framework.

**Electronic supplementary material:**

The online version of this article (10.1007/s00122-020-03716-8) contains supplementary material, which is available to authorized users.

## Introduction

A diallel experiment is based on a set of possible crosses between some homozygous (inbred) lines and it is usually aimed at quantifying genetic effects, such as:

1. *General combining ability (GCA)*, that is the discrepancy from the average performance of two parental lines in a hybrid combination. Based on Sprague and Tatum (1942), GCA mainly depends on the additive effects of genes, as well as on additive by additive interaction effects;
2. *Specific combining ability (SCA),* that is the effect by which certain hybrid combinations give relatively better/worse performances, compared to the average performances of their parental lines. SCA is regarded as an indication of loci with non-additive effects and includes dominance and epistatic interaction (additive by dominance and dominance by dominance interaction);
3. *Reciprocal (maternal) effect,* that relates to the discrepancy between the performances of a hybrid, e.g. ‘*A* × *B*’ and its reciprocal ‘*B* × *A*’. In some instances, (see Cockerham and Weir 1977), reciprocal effects are partitioned into two components: the *reciprocal general combining ability (RGCA)*, that refers, in general, to a parent line in all its combinations and the *reciprocal specific combining ability (RSCA)*, that refers to a specific combination of two parental lines. Reciprocal effects are of great importance for appropriate selection of parents as male or female in hybrid development (Mahgoub 2011);
4. *Heterosis*, that is the change in performance for crosses, with respect to parental lines.

The assessment of genetic effects is very useful in plant breeding; for example, a high GCA value can predict a flow of several desirable additive genes from parents to offspring (Franco et al. 2001). Moreover, the same authors showed that a high GCA estimate may indicate high heritability and low environmental effects, which may also result in low gene interactions, high selection response and large adaptability. It was also shown that GCA performances of future generations could be predicted by assessing the GCA of a line in an early generation (Lv et al. 2012), saving time and costs. On the contrary, for traits with non-additive gene action, selection should be undertaken in later generations, when genes will be fixed in the homozygous lines (Fasahat et al. 2015). Genetic effects were also determined to describe non-additive gene effects (Singh et al. 1986; Chigeza et al. 2014) and to identify heterotic groups or patterns (Napolitano et al. 2020).

Genetic effects may be of interest either by themselves or combined in the form of ratios. For example, the GCA/SCA ratio determines which type of gene action is involved: a relatively large ratio indicates the prevalence of additive genetic effects, while a relatively low ratio (e.g. < 1) the prevalence of dominance and/or epistatic gene effects (Christie and Shattuck 2010).

In order to estimate the above listed genetic effects, a diallel experiment may be planned by using four types of mating designs (Harriman and Nwammadu 2016), including:

1. Crosses, reciprocals and selfed parents (complete diallel)
2. Crosses and selfed parents (half-diallel: no reciprocals)
3. Crosses and reciprocals (no selfed parents)
4. Only crosses (no selfed parents, no reciprocals)

Furthermore, Griffing (1956) distinguished two different situations:

1. If the interest of the investigator lies only in the lines (parents) involved in the experiment and not beyond these crosses, the genetic effects are considered fixed;
2. If the lines (parents) are a sample from a wider population, the genetic effects are considered random and there is no interest in the effect of each single cross/parental line.

Considering the number of genetic effects, the four mating designs and the two possible models (fixed and random), we can easily understand the reasons why a plethora of estimation methods is available in literature, which may, at first, look overwhelming. For example, Hayman (1954) presented one method for mating design 1, while Griffing (1956) presented 8 different methods (4 mating designs, each with random and fixed effects). In Gardner and Eberhart (1966) two additional methods were introduced, later extended to include multiple environments (Eberhart and Gardner 1966).

What can be dreadful for a beginner is that all these estimation methods are not presented on a common ground. Furthermore, the choice of the appropriate method could be rather difficult, as not all of them can be interchangeably used in all situations. In fact, it has been shown that the estimates of SCA effects in Griffing’s methods 1 and 2 may be biased, due to the inclusion of parental lines (Yao et al. 2013). At the same time, Griffing’s method 3 was deemed to be the best for estimating SCAs and maternal/reciprocal effects. Nonetheless, Griffing’s method 4 is also considered reliable, it requires half of the crossings to be made, and hence, much less effort, especially when the number of parents is high (Acquaah 2012).

In this manuscript, following Möhring et al. (2011), we would like to reinforce the idea that all methods of analysis for diallel data should be seen as specific cases (different parameterisations) of a general linear model; consequently, all diallel models should be built and fitted by using a common platform.

Relating to random genetic effects, Möhring et al. (2011) put all models within the frame of linear mixed models (LMM) and restricted maximum likelihood (REML) estimation. They also proposed a software implementation with SAS and ASREML (Gilmoure et al. 2015). Other mixed model solutions have been proposed by Xiang and Li (2001), Wu and Matheson (2001), Xu and Zhu (1999). Focusing on the R statistical environment (R Core Team 2019), diallel mixed models can be efficiently fitted with the package ‘asreml-R’ (Butler et al. 2018) and with the free package ‘sommer’ (Covarrubias-Pazaran 2016). Several other possibilities exist, as listed in the introduction of the manuscript by Covarrubias-Pazaran (2016), while the most widespread packages for mixed models in R, i.e. ‘nlme’ (Pinheiro et al., 2020) and ‘lme4’ (Bates et al. 2015) do not appear to have been extensively used for fitting diallel models.

With concern to the fixed genetic effects, the traditional estimation methods proposed by Hayman (1954) and Griffing (1956) provide unbiased and minimum variance estimators only with balanced data. Furthermore, these estimation methods require specific patterns of calculations, whereas it would be desirable to have a general approach that also works for unbalanced data. The estimation of fixed genetic effects should be undertaken within the frame of general linear models, preferably by ordinary least squares (OLS), as already suggested by Gardner and Eberhart (1966). Implementations can be found for the SAS language (see Zhang and Kang 1997; Zhang et al. 2005; Makumbi et al. 2018) and in stand-alone software (Tong et al. 2012). To the best of our knowledge, the availability of tools for diallel analysis in the R statistical environment is rather limited. The already mentioned ‘asreml-R’ and ‘sommer’ packages are tailored to the needs of random effect estimation and only the latter is free to use. Other resources include the package ‘DiallelAnalysisR’ (Yaseen and Eskridge 2020) and the set of R functions presented in Singh et al. (2015). Both tools are self-contained and are not rooted in the typical frame of ordinary least squares (OLS) estimation in R.

Apart from REML (for mixed effects models) and OLS (for fixed effects models), a third option has recently made its way among geneticists, that is the Bayesian framework. Bayesian methods assume that, before making an experiments, model parameters are characterised by a prior probability distribution, that summarises our previous knowledge about the phoenomenon. After the experiment, the previous knowledge is updated by considering the observed data and it is expressed by way of a posterior distribution, representing our final knowledge about the phenomenon (Kery 2010). The Bayesian framework is very flexible and it can accommodate both random and fixed effects models, with several advantages in terms of interval estimation (Li and Loken 2002). Relating to diallel models, the use of a Bayesian frame for diallel models was advocated by Lenarcic et al. (2012), who created the R package ‘BayesDiallel’. An example of application in plant breeding was given by Turner et al. (2018).

It is somewhat surprising to see that, at present, fitting diallel models seems to require specific software and/or packages. In our view, all analyses should be performed by using standard, general purpose linear model fitting software. With focus on the R environment, it would not appear that, at present, fitting diallel models by using the most widespread functions, such as ‘lm()’, ‘lme()’ or ‘lmer()’ seems to be relatively rare. With reference to Bayesian methods, to the best of our knowledge there are no examples of using the very powerful and flexible BUGS environment (Spigelhalter et al*.* 2003).

This manuscript will initially focus on fixed genetical effects, relating the crosses between specific parents. The reason for this choice is three-fold: first, random/mixed effects diallel models can already be fitted in the general mixed model framework, by using the already mentioned ‘asreml-R’ package, or by using other general packages, such as ‘MCMCglmm’ (Hadfield 2010). Second, fixed effect models are useful to test for the significance of genetical effects, which is a common requirement among plant breeders. Last, but not least, while we recognise the flexibility of random effect models, we argue that the estimation of variance components may be unreliable when the number of parents is small. A brief survey of literature shows that mating designs with three (Amin 2015), four (Quimio and Zapata 1990) and five (Singh and Jain 1971; Dhaliwal and Gill 1973) parents are used. Furthermore, standard errors for variance components are seldomly reported; but, to our experience, they may be very high, also with mating designs with eight parents. Focusing on fixed genetic effects, the objective of the present work is to bridge the gap between linear models and diallel models in R. To do so, instead of building a brand new piece of software, we decided to follow another route, that is to build a set of tools which would enable geneticists, plant/animal breeders and students to fit diallel models by the most widely known R functions for OLS fitting. Finally, we will show that this set of tools can be also used to fit fixed/random effect diallel models in the Bayesian framework, by using a very widely known Markov Chain Monte Carlo (MCMC) sampler.

## Methods

### Model definitions

The results of diallel experiments might be described by using the usual two-way ANOVA model, where we consider the factorial combination of *n* parentals taken either as ‘father’ or as ‘mother’:

1 $$y_{ijk} = \mu + \gamma_{k} + \alpha_{i} + \beta_{j} + \alpha \beta_{ij} + \varepsilon_{ijk}$$

where *y*_*ijk*_ is the yield (or any other trait of interest) for the combination between the parents *i* and *j* in the block *k*, *μ* is the intercept, *γ*_*k*_ is the effect of the *k*th block, *α*_*i*_ is the ‘paternal’ effect of the *i*th ‘father’, *β*_*j*_ is the ‘maternal’ effect of the *j*th mother and *αβ*_*ij*_ is the interaction effect, describing the non-additive effect of a specific combination between the *i*th father and *j*th mother. The residual error term *ε*_*ijk*_ is assumed to be gaussian and i.i.d, with standard deviation equal to *σ*.

Except for the case of a full-diallel design, all the other diallel designs and so the one above is unbalanced; in all cases, the ‘father’ and ‘mother’ effects are regarded as two completely different series of treatments, neglecting the idea that they are, indeed, the same genotypes in different combinations. Therefore, there was the need to build more appropriate diallel models. For historical reasons, we will start from the model proposed by Hayman (1954), by slightly changing the notation, to make it more consistent, throughout the manuscript:

2 $$y_{ijk} = \mu + \gamma_{k} + g_{i} + g_{j} + ts_{ij} + rg_{i}^{a} + rg_{j}^{b} + rs_{ij} + \varepsilon_{ijk}$$

where *μ* is expected value (the overall mean, in the balanced case), *g*_*i*_ and *g*_*j*_ are the GCAs of the *i*th and *j*th parents, *ts*_*ij*_ is the total SCA (tSCA) for the combination between the *i*th and *j*th parent, $rg_{i}^{a}$ and $rg_{j}^{b}$ are the RGCAs for the *i*th and *j*th parents, under the constraint that $rg_{i}^{a}$ = $- rg_{j}^{b}$ for one specific parent *i* and *rs*_*ij*_ is the RSCA for a specific *ij* combination, i.e. the discrepancy between the effect of the *i* and *j* parents, when they are used as ‘father’ or ‘mother’, respectively. Obviously, reciprocal effects can only be estimated when the experimental design includes the reciprocal crosses.

The four models devised by Griffing (1956) need not be listed, as they can be seen as particular cases of the more general Eq. 2, where the reciprocal effect (REC) is not parted into RGCA and RSCA $\left( {r_{ij} = rg_{i}^{a} + rg_{j}^{b} + rs_{ij} } \right)$. Of course, when the reciprocals have not been included in the mating design, the term $r_{ij}$ should be removed from the equation.

According to Hayman (1954), the tSCA effect can be partitioned in three additive components, leading to the following system of equations:

3 $$y_{ijk} = \left\{ {\begin{array}{*{20}l} {\mu + \gamma_{k} + g_{i} + g_{j} + m + d_{i} + d_{j} + s_{ij} + rg_{i}^{a} + rg_{j}^{b} + rs_{ij} + \varepsilon_{ijk} } \hfill & {{\text{for}}\;i \ne j} \hfill \\ {\mu + \gamma_{k} + 2g_{i} - \left( {n - 1} \right)m - \left( {n - 2} \right)d_{i} + \varepsilon_{ijk} } \hfill & {{\text{for}}\;i = j} \hfill \\ \end{array} } \right.$$

where *n* is the number of parentals, *m* relates to the difference between the average yield of selfed parents and the average yield of crosses (mean dominance deviation; MDD), the *d* parameters relate to the differences between the yield of each selfed parent (*Y*_*ij*_, with *i* = *j*) and the average yield of all selfed parents (dominance deviation for the *i*th parent; DD) and *s*_*ij*_ is the residual SCA effect for the combination *ij*.

It should be noted that both Eqs. 2 and 3 consider the genetical effects as differences with respect to the intercept *μ*, that is the mean of all observations. Due to unbalance (the number of crosses is never equal to the number of selfed parents), such an approach requires the introduction of some coefficients (i.e. *n* − 1 and *n* − 2 in Eq. 3), which do not have an obvious meaning. Additional models were proposed, which do not consider the overall mean as the intercept, but allow for different means for crosses and selfed parents (Gardner and Eberhart 1966). One such model is usually known as GE2, and it may be formulated as:

4 $$y_{ijk} = \mu_{\nu } + \gamma_{k} + 0.5\left( {v_{i} + v_{j} } \right) + \overline{h} + h_{i} + h_{j} + s_{ij} + \varepsilon_{ijk}$$

where *μ*_*v*_ is the intercept, corresponding to the overall mean for all selfed parents (not the overall mean, as in previous models). The parameters *v* (*v*_*i*_ and *v*_*j*_) represent the differences between the expected value for the selfed parents *i* and *j* and the mean for all selfed parents (*μ*_*v*_). According to the authors, this would be the variety effect (VE); as a consequence, the expected value for the *i*th selfed parent is *μ*_*v*_ + *v*_*i*_, while the expected value for the cross *ij*, in the absence of any dominance/heterosis effects, would be *μ*_*v*_ + 0.5(*v*_*i*_ + *v*_*j*_), that is the mean value of its parents. There is a close relationship between *g*_*i*_ and *g*_*j*_ in Eqs. 2 and 3 and *v*_*i*_ and *v*_*j*_ in Eq. 4, that is: *v*_*i*_ = 2*g*_*i*_ + (*n* − 2)*d*_*i*_; therefore, the sum of squares for the GCA and VE effects are the same, although the estimates are different.

Since a cross not necessarily responds according to the mean value of its parents, the parameter $\overline{h}$ represents the average heterosis (H.BAR) contributed by the whole set of genotypes used in crosses. In the balanced case, $\overline{h}$ represents the difference between the overall mean for selfed parents and the overall mean for crosses, under the constraint that $\overline{h} = 0$ for all selfed parents. Besides, the parameters *h*_*i*_ represent the average heterosis contributed by the *i*th parent in its crosses (Hi), while *s*_*ij*_ is the SCA for the cross between the *i*th and *j*th parents, that is totally equivalent to the corresponding parameter in Eq. 3.

It is clear that both Eqs. 3 and 4 account for the heterosis effect, although they do it in a different way: in Eq. 3 the specific effect of heterosis is assessed with reference to the overall mean, while in Eq. 4 it is assessed by comparing the mean of a cross with the means of its parents. Indeed, the sum of squares for the ‘MDD’ and ‘Hi’ effects are perfectly the same, although the parameters are different.

Gardner and Eberhart proposed another model (GE3), which we have slightly modified to maintain a consistent notation in the frame of GLMs:

5 $$y_{ijk} = \left\{ {\begin{array}{*{20}l} {\mu_{\nu } + \gamma_{k} + \overline{h} + {\text{gc}}_{i} + {\text{gc}}_{j} + s_{ij} } \hfill & {{\text{for}}\;\;i \ne j} \hfill \\ {\mu_{\nu } + \gamma_{k} + {\text{sp}}_{i} } \hfill & {{\text{for}}\;\;i = j} \hfill \\ \end{array} } \right.$$

Equation 5 is an array composed of two separate elements for crosses and selfed parents. For the crosses (equation above), the parameters $gc_{i}$ and $gc_{j}$ represent the GCA for the *i* and *j* parents in all their crosses (GCAC); it should be noted that GCA ≠ GCAC, as this latter effect is estimated without considering the selfed parents. The parameters *s*_*ij*_ are the same as in the previous models (SCA effect), while *sp*_*i*_ represents the effects of selfed parents (SP): they are numerically equivalent to the corresponding effects in Eq. 4, but the sum of squares are different and such a discrepancy has been put forward and discussed by Murray et al. 2003, to whom we refer for further detail. Therefore, we use different names for these two effects (SP and Hi).

### Model implementation

Our view is that all diallel models (Eqs. 2 to 5 and other similar equations) are linear models with different parameterisations. Therefore, it would be useful to be able to fit all those models in R, by using the standard, general purpose facilities for linear model fitting. Focusing on fixed effects, every linear model can be written (in matrix notation) as:

6 $$y = X \beta + \varepsilon$$

where *y* is the vector of the observed response, *X* is the design matrix, *β* is the vector of parameters and *ε* is the vector of residuals, assumed as gaussian distributed, with mean equal to 0 and variance equal to *σ*^2^. The estimation of β is accomplished by minimising the sum of squared residuals (RSS = *ε*^T^*ε*), which is possible by the following equation:

7 $$\beta = (X^{T} X)^{ - 1} X^{T} y$$

The OLS solution is also the maximum likelihood solution, provided that errors are independent and identically distributed. We see that Eq. 7 requires the availability of the design matrix *X*.

In R, the typical linear model fitting function based on OLS is ‘lm()’, which uses the ‘model.matrix()’ function to build design matrices, according to the user-defined (or default) parameterisation. The main implementation problem is that certain effects, such as the GCA, require the definition of unconventional design matrices, using algorithms that are not available in R. The packages ‘asreml-R’ and ‘sommer’ add a few functionalities, such as the ability of overlaying design matrices (function ‘and()’ in ‘asreml’ and ‘overlay()’ in ‘sommer’), which is useful to code GCA effects. However, none of the two packages plays well with the ‘lm()’ function in R and at present, to the best of our knowledge, there is no simple way to fit diallel models with fixed effects by using the ‘lm()’ function in R.

Several authors suggested how to build design matrices for half-diallel designs (Wu and Matheson 2000, 2001; Tong et al. 2012). We extended these suggestions to build a handful of new R functions, aimed at producing the correct design matrices for all the above mentioned effects. The syntax is simple; for example, the GCA effect can be specified by using the function ‘*GCA(Par1, Par2)*’, where ‘Par1’ and ‘Par2’ are two variables coding for parentals. For all other effects, only the name of the function changes, according to the naming in previous paragraphs (GCA, tSCA, RGCA, RSCA, REC, DD, MDD, H.BAR, Hi, VEi, SP and GCAC).

By using these R functions, we can fit all diallel models inside the ‘lm()’ and ‘lme()’ functions. For example, Eq. 3 can be fitted by using the usual code for linear models:

$${\text{lm}}\left( {{\text{yield}}\sim{\text{GCA}}\left( {{\text{Par}}1,\,{\text{Par}}2} \right) + {\text{tSCA}}\left( {{\text{Par}}1,\,{\text{Par}}2} \right),\,{\text{data}} = {\text{df}}} \right)$$

where ‘df’ is a ‘dataframe’ hosting the response and explanatory variables. Similarly, we can introduce the effect of reciprocals by using the following code:

$${\text{lm}}({\text{yield}}\sim{\text{GCA}}\left( {{\text{Par}}1,\,{\text{Par}}2} \right) + {\text{tSCA}}\left( {{\text{Par}}1,\,{\text{Par}}2} \right) + {\text{REC}}\left( {{\text{Par}}1,\,{\text{Par}}2} \right),\,{\text{data}} = {\text{df}})$$

This latter definition corresponds to Griffing’s model 1 (Eq. 2, replacing $rg_{i}^{a} + rg_{j}^{b} + rs_{ij}$ with *r*_*ij*_); however, if we were willing to partition the tSCA effect, we could code the following model:

$$\begin{gathered} {\text{lm}}({\text{yield}}\sim{\text{GCA}}\left( {{\text{Par}}1,\,{\text{Par}}2} \right) + {\text{MDD}}\left( {{\text{Par}}1,\,{\text{Par}}2} \right) + {\text{DD}}\left( {{\text{Par}}1,\,{\text{Par}}2} \right) \hfill \\ + {\text{SCA}}\left( {{\text{Par}}1,\,{\text{Par}}2} \right) + {\text{REC}}\left( {{\text{Par}}1,\,{\text{Par}}2} \right),\,{\text{data}} = {\text{df}}) \hfill \\ \end{gathered}$$

which does not correspond to any of the Hayman’s, Griffing’s or Gardner–Eberhart’s models, nonetheless it has relevant potentialities. If we replace ‘*REC(Par1, Par2)’* with ‘*RGCA(Par1, Par2)* + *RSCA(Par1, Par2)’* we get Hayman’s model 2 (Eq. 3); in case of no reciprocals, we can remove the REC effects altogether, while in case of no reciprocals and no selfed parents, we can build a model with the GCA effect only.

Another possible model is:

$${\text{lm}}({\text{yield}}\sim{\text{H.BAR}}\left( {{\text{Par}}1,\,{\text{Par}}2} \right) + {{\text{VE.i}}} \left( {{\text{Par}}1,\,{\text{Par}}2} \right) + {\text{H.i}}\left( {{\text{Par}}1,\,{\text{Par}}2} \right) + {\text{SCA}}\left( {{\text{Par}}1,\,{\text{Par}}2} \right),\,{\text{data}} = {\text{df}})$$

that corresponds to Gardner–Eberhart model 2 (Eq. 4) and could be enhanced by including the effects ‘*REC(Par1, Par2)’* or ‘*RGCA(Par1, Par2)* + *RSCA(Par1, Par2)*’, when reciprocals are available. Lastly, the GE3 model (Eq. 5) is:

$${\text{lm}}({\text{yield}}\sim{\text{H}}.{\text{BAR}}\left( {{\text{Par}}1,\,{\text{Par}}2} \right) + {\text{SP}}\left( {{\text{Par}}1,\,{\text{Par}}2} \right) + {\text{GCAC}}\left( {{\text{Par}}1,\,{\text{Par}}2} \right) + {\text{SCA}}\left( {{\text{Par}}1,\,{\text{Par}}2} \right),\,{\text{data}} = {\text{df}})$$

that can, as well, be enhanced by adding reciprocal effects, if necessary.

In summary, we propose that diallel models are flexibly built by using the typical process of model fitting that has become in fashion with GLMs, considering: (i) the information we have at hand (whether we have crosses, selfs and/or reciprocals) and (ii) the effects we want to estimate. In this process, we have only one model frame and different parameterisations, as anticipated above.

However, we do not intend to neglect the importance of referring to some relevant model parameterisations, by using the names of the authors. For this reason, we also built a wrapper function named ‘lm.diallel()’, which can be used in the very same fashion as ‘lm()’. The syntax is:

$${\text{lm.diallel}}\left( {{\text{formula}},\,{\text{Block}},\,{\text{Env}},\,{\text{data}},\,{\text{fct}}} \right)$$

where ‘formula’ uses the regular R syntax to specify the response variable and the two variables for parentals (e.g., Yield ~ Par1 + Par2). The two arguments ‘Block’ and ‘Env’ are used to specify optional variables, coding for blocks and environments, respectively. The argument ‘data’ is a ‘dataframe’ where to look for explanatory variables. Finally, ‘fct’ is a string variable coding for the selected model. In this regard, we considered the main six diallel models in literature: Hayman’s model 1 (Eq. 2), Hayman’s model 2 (Eq. 3), Griffing’s model 1 (Eq. 2, with $r_{ij} = rg_{i}^{a} + rg_{j}^{b} + rs_{ij}$), Griffing’s model 2 (Eq. 2, without reciprocal effects), Gardner–Eberhart model 2 (Eq. 4) and Gardner–Eberhart model 3 (Eq. 5). For these six models, the ‘fct’ string should take, respectively, the following values: “HAYMAN1”, “GRIFFING1”, “GRIFFING2”, “HAYMAN2”, “GE2”, “GE3”. The strings “GE2r” and “GE3r” can be used to specify the ‘enhanced’ GE2 and GE3 models, including the effect of reciprocals (REC).

As an example, the GE3 model can be fitted either by using ‘lm()’ (as shown above) or by using the following syntax:

$${\text{lm.diallel}}\left( {{\text{yield}}\sim{\text{Par}}1 + {\text{Par}}2,\,{\text{data}} = {\text{df}},\,{\text{fct}} = {\text{``GE3"}}} \right)$$

For better clarity, we report a table of the correspondences between the equations, the syntax for the ‘lm()’ function and the value for the ‘fct’ argument in the ‘lm.diallel()’ function (Table 1).

**Table 1:** Correspondence between the Eqs. 2 to 5, the value for the ‘fct’ string in the ‘lm.diallel()’ function and the syntax for the ‘lm()’ function

Equation	Model name in 'lm.diallel()'	Model notation in 'lm()'
Equation 2	HAYMAN1	Y ~ GCA(Par1, Par2) + tSCA(Par1, Par2) + RGCA(Par1, Par2) + RSCA(Par1, Par2)
Equation 2^a^	GRIFFING1	Y ~ GCA(Par1, Par2) + tSCA(Par1, Par2) + REC(Par1, Par2)
Equation 2^b^	GRIFFING2	Y ~ GCA(Par1, Par2) + tSCA(Par1, Par2)
Equation 3	HAYMAN2	Y ~ GCA(Par1, Par2) + MDD(Par1, Par2) + DD(Par1, Par2) + SCA(Par1, Par2) + RGCA(Par1, Par2) + RSCA(Par1, Par2)
Equation 4	GE2	Y ~ H.BAR(Par1, Par2) + VE.i(Par1, Par2) + H.i(Par1, Par2) + SCA(Par1, Par2)
Equation 5	GE3	Y ~ H.BAR(Par1, Par2) + SP(Par1, Par2) + GCAC(Par1, Par2) + SCA(Par1, Par2)

^a^Terms are redefined as: $r_{ij} = rg_{i}^{a} + rg_{j}^{b} + rs_{ij}$

^b^The term *r*_*ij*_ is not included

One big advantage of fitting diallel models in R with the ‘lm()’ function or with the ‘lm.diallel()’ function is that we can exploit the whole infrastructure for ‘lm’ objects. In particular, we can make profit, e.g. of the usual ‘plot’, ‘summary’ and ‘anova’ methods. With reference to these latter two methods, we should not forget that, by default, the residual term is used as an estimate of pure error. Although the genetic effects in our approach are fixed, using the residual error term is not necessarily a good way to go. For example, the residual error term may not be available, if we work with means and not with raw field data. Furthermore, if we have collected data about the reciprocals, but we do not want to fit reciprocal effects, the residual term is not a good estimate of pure error.

Therefore, we have coded the ‘anova.diallel’ and ‘summary.diallel’ methods that, optionally, allow the user to enter a value for the residual error variance and degrees of freedom. This user-defined value is used in inferences and tests of hypotheses, in place of the residual term.

### Linear/nonlinear functions of model parameters

One problem with diallel models is that the effects may not be orthogonal to each other, which may cause some inconsistencies in estimation (Murray et al. 2003). Therefore, it has been proposed that some meaningful quantities (the forementioned authors cite ‘heterosis effects’, ‘variety effects’ and GCA) are directly derived from variety and cross means. In the frame of linear models, it is possible to derive those meaningful quantities by linear and nonlinear functions of model parameters (Bretz et al. 2011), while standard errors can be derived by using the propagation of errors and the delta method (Weisberg 2005). In simple terms, when we combine the values of model parameters to derive some meaningful quantity, e.g. an index of heterosis, we can also combine the standard errors to derive a standard error for that heterosis index. If the function of model parameters is linear, the derived standard error is exact; otherwise, it is only approximate and takes the name of delta standard error. With specific reference to ratios, the Fieller's method can also be used for inferences (Piepho and Emrich 2005).

For example, the parameters of Eq. 4 can be easily used to derive mid-parent heterosis (MPH) and best parent heterosis (BPH) (Li et al. 2018). The two equations are:

8 $${\text{MPH}}\left( \% \right) = \frac{{\overline{h} + h_{i} + h_{j} + s_{ij} }}{{\mu_{\nu } + 0.5 \times \left( {v_{i} + v_{j} } \right)}}$$

and:

9 $${\text{BPH}}\left( \% \right) = \frac{{\overline{h} - 0.5 \times \left( {v_{i} + v_{j} } \right) + h_{i} + h_{j} + s_{ij} }}{{\mu_{\nu } + \max \left( {v_{i} ,v_{j} } \right)}}$$

Other useful measures can be derived in the very same fashion, as linear or nonlinear functions of model parameters.

### Extension to random and mixed models

Although we have so far focused on fixed effects, we do not neglect the importance and flexibility of building models with random genetic effects. A multilevel mixed model with *r* random terms can be written in matrix notation as:

10 $$y = X \beta + Z_{1} b_{1} + Z_{2} b_{2} + \ldots + Z_{r} b_{r} + \varepsilon$$

where *Z*_1_, *Z*_2_ and *Zr* are the design matrices for random effects and *b*_1_, *b*_2_, *b*_*r*_ are the vectors of random effects, which are assumed to be independent from each other, independent from the residual error term and gaussian distributed, with means equal to 0 and variances respectively equal to *σ*^2^_*b*1_, *σ*^2^_*b*2_ and *σ*^2^_*b*r_ (variance components). In contrast to Eqs. 6, 10 has no immediate closed form solution and therefore, parameter estimates need to be obtained by using some sort of mixed model “solver”, such as those available in the EMMREML (Akdemir and Godfrey 2015) and SAMM (Akdemir 2018) packages, just to mention a few. These solvers require the design matrices for fixed and random terms (*X*, *Z*_1_, *Z*_2_, …, *Z*_*r*_), which can be built by using the forementioned tools, together with the ‘model.matrix()’ function. We also built the ‘model.matrixDiallel()’ functions that is simpler to use and shares the same syntax as the 'lm.diallel()' function.

For one of our examples, we used the very popular, general purpose MCMC sampler JAGS with its companion R package ‘rjags’ (Plummer 2019), which appear to be underutilised in the context of plant breeding, although they are very powerful and flexible. JAGS is rooted in the Bayesian framework and requires prior information about all the estimands. For fixed effects, we used Gaussian priors with means equal to 0 and precisions equal to 0.0001 (the precision is the inverse of the variance and it is used in place of this latter in the definition of mixed models in JAGS). For variance components, we used uniform priors from 0 to 100. The MCMC sampler was used with five chains, 10,000 iterations and a burn-in of 1000 iterations.

### R package

All the above R functions and JAGS model definitions are freely available in a gitHub repository (https://github.com/OnofriAndreaPG/lmDiallel) and they have been used to produce an R package (lmDiallel), which can be freely downloaded from gitHub (the code download and installation are shown below). They were written by the standard R language, and they depend on the basic packages ‘base’ and ‘stat’, as well as on the package ‘plyr’ (Wickham 2011). The engine for the JAGS sampler also needs to be installed, together with the ‘rjags’ package.

## Results

The functionalities and approach of our package can be better described by using a few examples, relating to different experimental situations.

### *Example 1*

As an example of mating design 1, we used the dataset shown in Hayman (1954), concerning the flowering times in *Nicotiana rustica*, in a diallel cross with eight inbred varieties. The design was a randomized complete block design with two replicates and for this analysis, we will regard the block as fixed effect. The dataset is available in the ‘lmDiallel’ package as ‘hayman54’.

Relating to the code in Box 1, the first three lines install (if necessary) and load the ‘lmDiallel’ package from gitHub (the ‘devtool’ package is necessary to perform the installation and it must have been already installed in the system). The forth line loads the dataset and the subsequent lines fit the Eq. 2 (model HAYMAN2) and show the ANOVA table. The results are entirely the same as those reported in the original paper (see also the supplemental material in Möhring et al. 2010). Being in the typical R platform for linear models has the great advantage that we can use most of the available methods for linear models. Apart from the ‘anova()’ method, we can also use the ‘summary()’ method to retrieve the genetic parameters, as commonly done for linear models in R. In Box 2, we show an excerpt of the output.

**Box 1:**
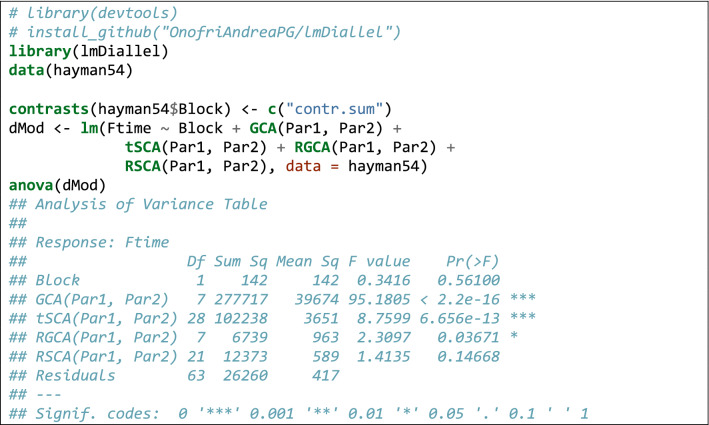
Sample code to fit Eq. 2 to the data in Hayman (1954)

From Box 2, we see that some estimates are missing. Indeed, we should not forget that parameter estimation is performed under some restrictions, e.g. $\sum\nolimits_{i} {j_{i} } = 0$ and $\sum\nolimits_{i} {k_{i} } = 0$, therefore, $j_{8} = - \sum\nolimits_{i = 1}^{7} {j_{i} }$ and $k_{8} = - \sum\nolimits_{i = 1}^{7} {k_{i} }$. Linear functions of model parameters can be built by using the standard facilities in R, such as the ‘glht()’ function in the ‘multcomp’ package (Bretz et al. 2011). An example is given in Box 3.

**Box 2:**
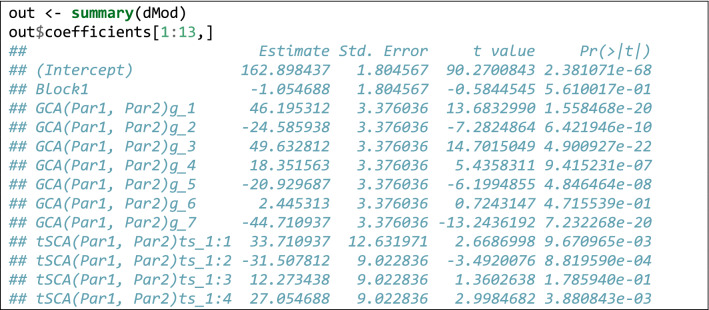
Genetical parameters for the data in Hayman (1954)

**Box 3:**
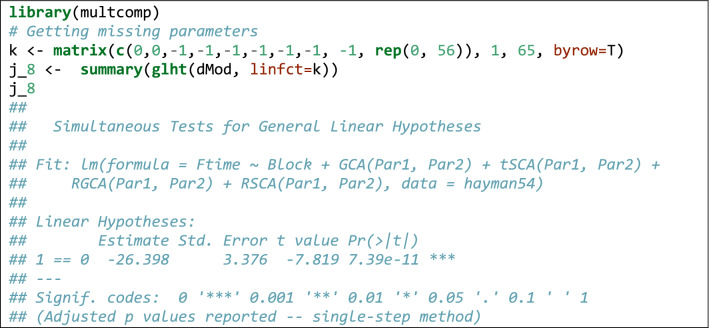
Use of the *glht()* function in the *multcomp* package to build linear functions of model parameters

**Fig. 1:**
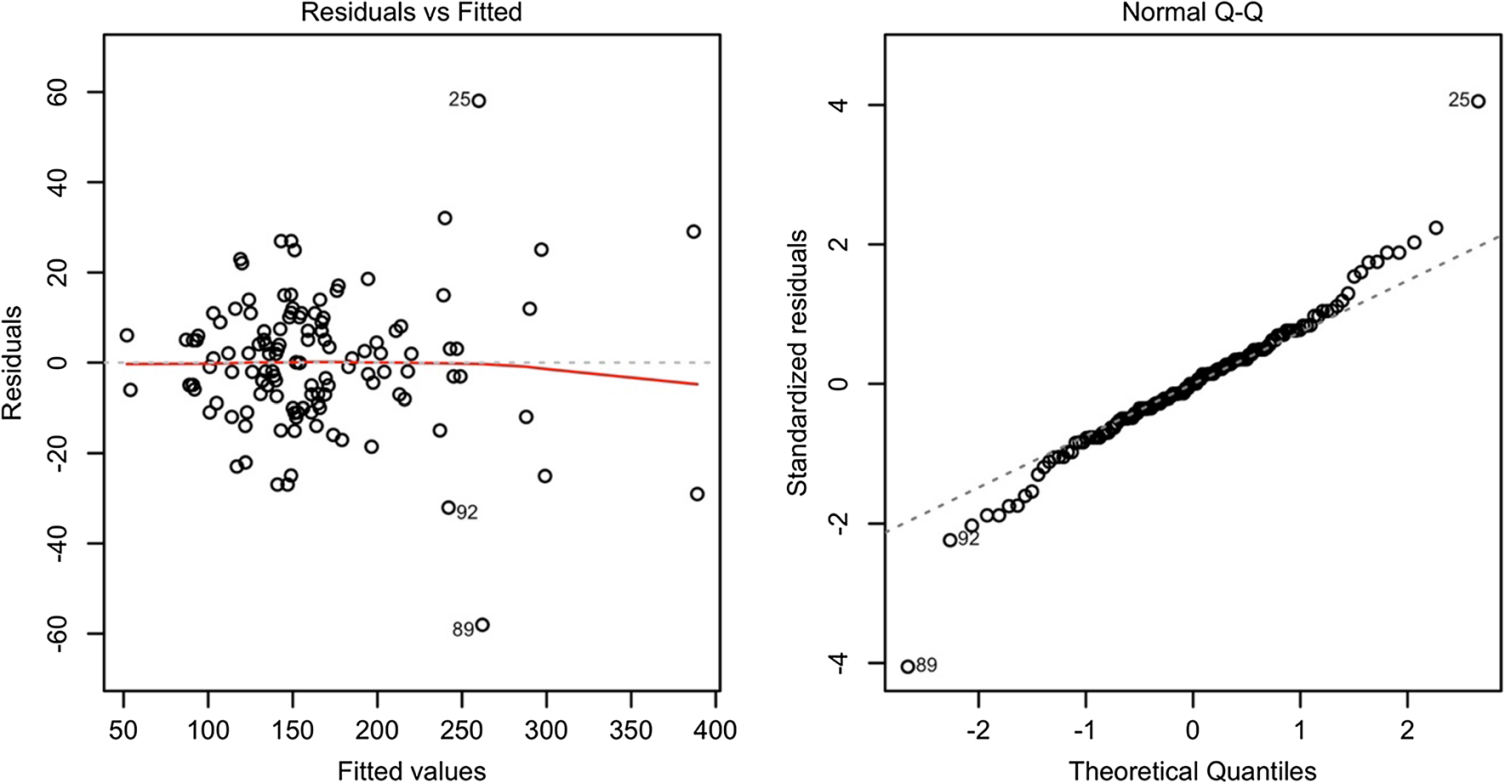
Graphical inspection of residuals for a diallel model: plot of residuals against expected values (left) and QQ-plot of standardised residuals (right)

A frequently neglected aspect is that diallel models, as all linear models, should be carefully inspected in relation to the basic assumptions of normality and homoscedasticity of residuals. A graphical inspection can be obtained with the usual ‘plot.lm()’ method, the result is shown in Fig. 1. We see some slight signs of heteroscedasticity, which we will not address here, although we would like to mention that such a problem might be solved by using stabilising transformations or appropriately modelling the variance–covariance matrix for model residuals (Pinheiro and Bates 2000). The selection of the most appropriate method is still under debate, and both solutions have advantages and drawbacks. We refer the readers to the available literature for multi-environment experiments (e.g. Annicchiarico 2002), and we only emphasize that the inspection of model residuals appears to be as fundamental for diallel models as it is for all other linear models.

An alternative, though less flexible, way of fitting the same model is by using the wrapper function ‘lm.diallel()’, which is shown in Box 4. In this case, we do not have to specify the effects, we only have to indicate what model we want to fit (see also Table 1).

**Box 4:**
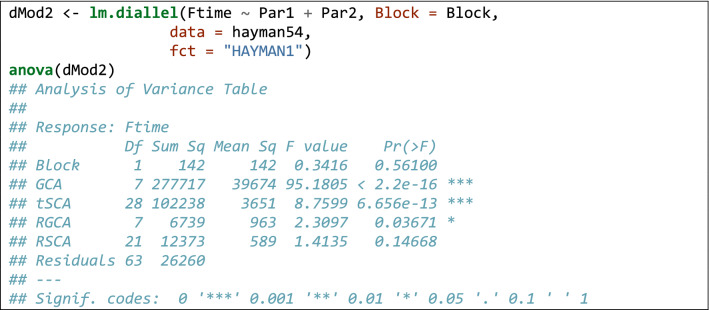
Use of the *lm.diallel()* wrapper to fit the same model as in Box 1

### *Example 2*

Very often, diallel experiments are repeated across environments (different locations and/or different years). The method outlined in the previous example can be used to produce separate analyses for each environment. However, if the environment is regarded as a fixed factor, we could be interested in testing the significance of the genetical effects and their interactions with the environment.

In order to show how this can be performed in R, we used the dataset shown in Zhang et al. (2005), reporting the results of a diallel experiment with five parents, in two blocks and two environments. Reciprocal crosses are not considered, and they were deleted from the dataset. The dataset (‘Zhang05’ in Box 5) contains the ‘Par1’, ‘Par2’, ‘Env’, ‘Block’ and ‘Yield’ variables, coding for the parents, environments, blocks and yield, respectively. Box 5 shows the code to fit Eq. 4.

**Box 5:**
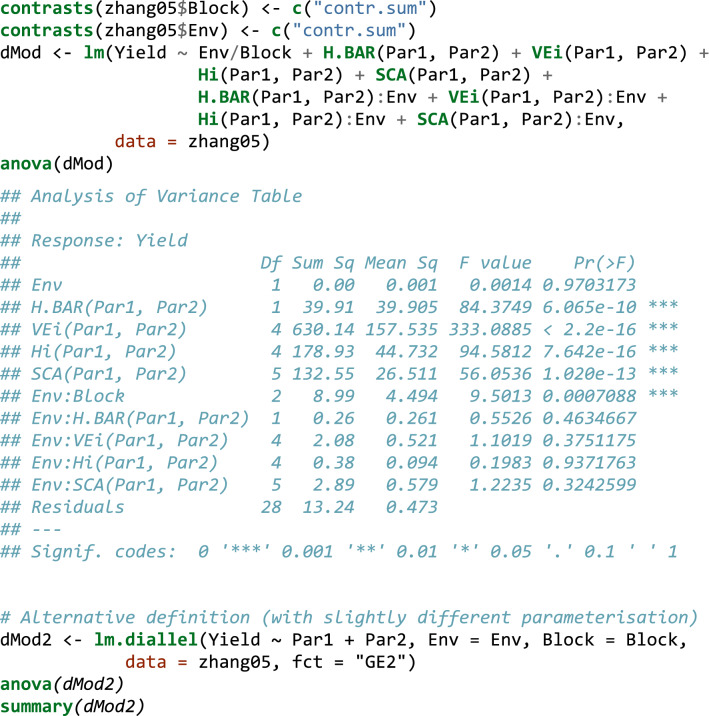
Example of how diallel models can be fit by using the ‘lm()’ function in R.

The ANOVA table shows that there are no significant interactions between genetical parameters and the environment. Therefore, we can remove those interactions and refit the model to get the average value of genetical parameters.

Functions of model parameters can also be used to retrieve the MPH (%) and BPH (%) (see Eqs. 8 and 9), together with standard errors. In this respect, we can use the ‘deltaMethod()’ function in the ‘car’ package (Weisberg 2005). Box 6 gives a simple example for the ‘1 × 2’ cross, which can be easily extended to other crosses by an appropriate script.

**Box 6:**
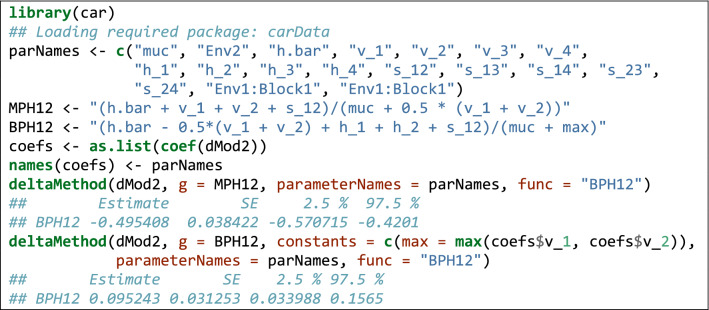
Using the delta method to retrieve MPH and BPH with standard errors.

Once again, we see that all analyses can be performed by the typical facilities in R, with a little experience in linear modelling. We also like to emphasise that in Box 7 we have removed the environment effects altogether, although in other instances, we might be interested in removing the interaction with the environment only for a subset of genetical effects.

**Box 7:**
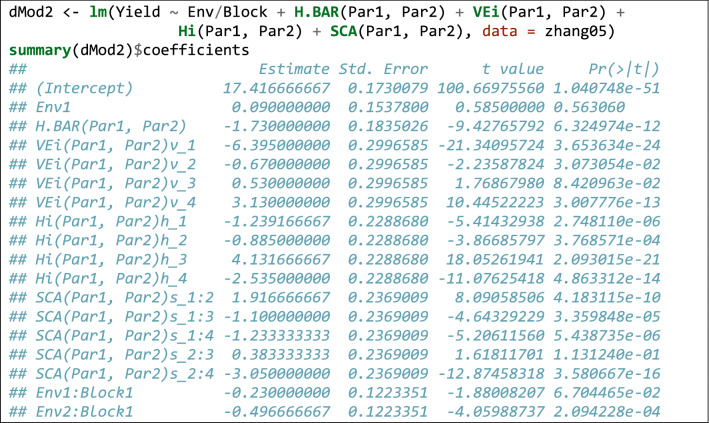
Removing the environment effect from the model in Box 5.

### *Example 3*

This dataset is from a diallel experiment with six maize varieties and no reciprocals (Gardner and Eberhart 1966) and consists of means across blocks, a possibility when we perform the analyses in two-steps: firstly, running separate analyses for all locations and secondly, fitting a model to the entry means. In order to perform the correct inferences, we need an estimate of an appropriate error term, that is usually obtained in the first step; in this example, we used the residual variance (MSE = 7.10 with 60 degrees of freedom), as reported in the paper.

The dataset is available as ‘lonnquist61’ in the ‘lmDiallel’ package. The code shown in Box 8 is used to fit a model with GCA effects retrieve the value of estimated parameters. Please, note that the residual variance is passed as an argument to the ‘summary()’ function, to obtain reliable estimates of standard errors. The estimated parameters and the partitioning of sum of squares are entirely the same as those reported in the paper.

**Box 8:**
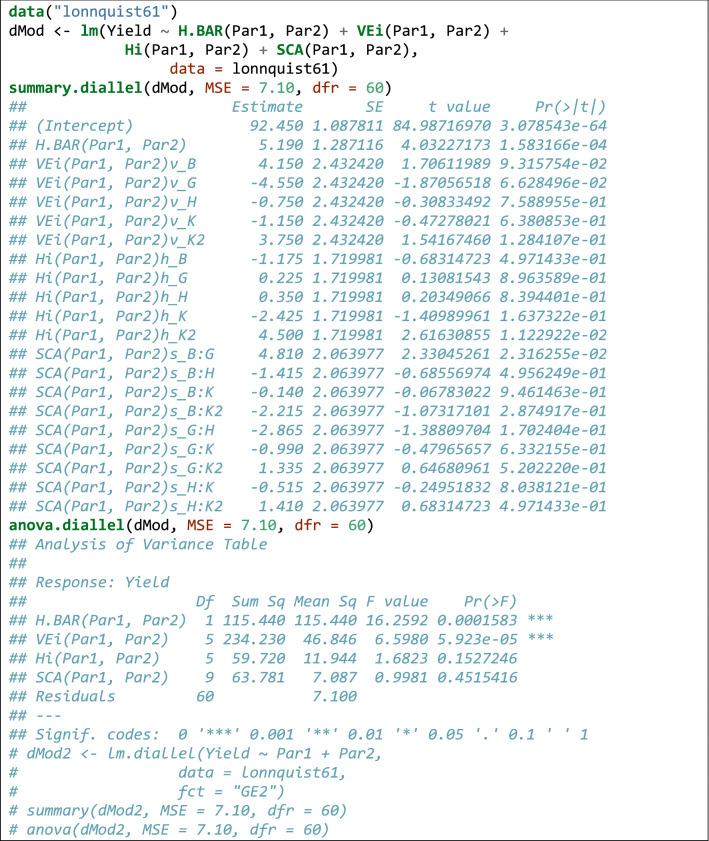
Sample code to fit Eq. 4 to the data in Gardner and Eberhart (1966), either with the ‘lm()’ function or with the ‘lm.diallel()’ wrapper.

### *Example 4*

(**extension to mixed models**) For this final example, we used a multi-environment half-diallel dataset with six parentals, in five blocks and ten environments; the dataset is factitious and was obtained by Monte Carlo simulation, starting from the data shown in Example 2. It is available in the 'lmDiallel' package in the 'diallelMET' data object. We want to fit the Gardner–Eberhart model 2 and as we have a relatively high number of blocks and environments, we are willing to consider these two effects as random. Extending Eq. 10, the model is:

11 $$\left\{ {\begin{array}{*{20}l} {y = X\beta + Z_{1} b_{1} + Z_{2} b_{2} + Z_{3} b_{3} + Z_{4} b_{4} + Z_{5} b_{5} + Z_{6} b_{6} + \varepsilon } \\ {\varepsilon \sim N\left( {0,\sigma } \right)} \\ \end{array} } \right.$$

where *X* is the design matrix for fixed effects, *β* is the vector of fixed effects; *ε* is the vector of model residuals, that is assumed as normally distributed with mean equal to 0 and standard deviation equal to *σ*; the number of columns in *X* is equal to the number of elements in *β*, that is 21 (with five parentals: one column/element for the interaction effect, one for the H.BAR effect, five for the VE.i effect, five for the H.i effect and nine for the SCA effect). The six *Z*_*i*_ matrices and *b*_*i*_ vectors are, respectively, the design matrices for the random effects of ‘environments’ (*Z*_1_*b*_1_, with 9 columns/elements), ‘blocks within environments’ (*Z*_2_*b*_2_, with 40 columns/elements), ‘H.BAR by environments’ (*Z*_3_*b*_3_, with 9 columns/elements), ‘VE.i by environments’ (*Z*_4_*b*_4_, with 45 columns/elements), ‘H.i by environments’ (*Z*_5_*b*_5_, with 45 columns/elements) and ‘SCA by environments’ (*Z*_6_*b*_6_, with 81 columns/elements). All random effects are assumed as gaussian, with means equal to 0 and standard deviations equal to $\sigma_{1} ,\,\sigma_{2} ,\,\sigma_{3} ,\,\sigma_{4} ,\,\sigma_{5} \;{\text{and}}\;\sigma_{6} ,$ respectively. As specified in Eq. 11, the residual term *ε* is also assumed as gaussian, with mean equal to 0 and standard deviation *σ*. As the consequence, there are 28 estimands.

In order to fit the above model with JAGS, we have to specify the definition (in JAGS code) in a text file, as shown in the Supporting Information S1. This definition should contain the likelihood expression for the model to be fitted (Eq. 11), the priors for all estimands (*β* and *b*_1_ to *b*_6_) and the hyperpriors for the standard deviation parameters (*σ* and *σ*_1_ to *σ*_6_). The definition of a JAGS model may require some experience; to ease the task, we provide several template files as a list in the ‘bugs_mods’ dataset, which can be loaded in R and saved to a text file within the working directory. In Box 9, we show how to load the dataset and JAGS model definition, saving this latter to the ‘modelDef.txt’ file, to be used in subsequent steps.

**Box 9:**
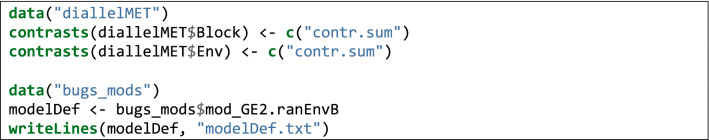
Loading the dataset and model definition.

Now, we need to create the design matrices, which is feasible by using the ‘model.matrix()’ function and dismantling the resulting object by way of the ‘assign’ attribute. We need to know that such an attribute labels the columns belonging to each effect in the model, according to their positioning, from left to right (see Box 10). We also need starting values for the estimands; for fixed effects, we can use the results of a fixed model fit, while for random effects we can set the starting values to 0.1. Care should be taken to ensure that the naming of parameters in the JAGS call correspond to their naming within the model definition file. In the end, we can start the MCMC sampler to obtain samples from the posterior distribution for all the estimated parameters. For the random effects, we request samples for the variance components, although the model was parameterised in terms of the standard deviations.

**Box 10:**
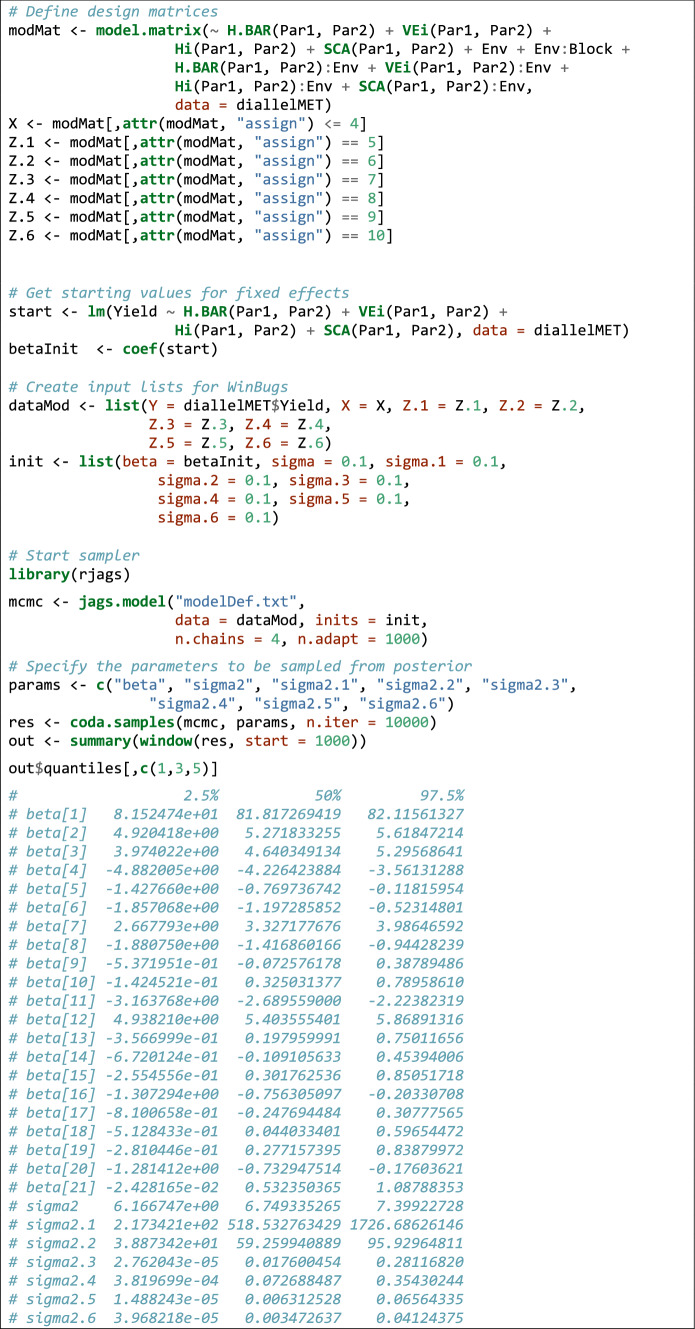
R code to fit a GE2 model to a multi-environment diallel experiment, with random environments and blocks.

The model may take some time to fit: in the end, for all estimated parameters we obtain information about the posterior distribution, i.e. the median as measure of central tendency and the credible interval, which is the Bayesian analogue to the confidence interval, although the interpretation is rather different (Onofri 2015). The whole code to perform the analyses is shown in Box 10, where for brevity reasons, we omitted the report about random effects and we only show variance components. Random effects can be simply obtained by appropriately changing the ‘init’ and ‘params’ variables in model definition.

## Discussion

Diallel experiments are frequently used to obtain information about the genetical parameters of parental lines. A survey of literature shows that, starting from the mid of the previous century, several models and methods have been proposed, which may be overwhelming for a novice. The most confusing aspect is that all those models and methods are not presented on a common platform, which enhances the perceived difference from one model to another. In this regard, we have tried to reinforce the idea that all diallel models are special cases (different parameterisations) of the same general linear model (see also Möhring et al. 2011).

In order to promote the above view, we have argued that it should be possible to fit diallel models by using the typical frame of OLS estimation, with no need for additional fitting tools. So far, software development has followed the route to building specific tools for diallel analyses; we thought that, instead of building brand new functions for diallel models, it would be relevant to give geneticists and plant/animal breeders the tools to fit diallel models within the general platform of linear models in R.

Linear fixed effect models in R are mainly fitted by using the ‘lm()’ function and related methods, although at the moment, diallel models cannot be fit by using such function. The problem is that there are no tools to automatically build the design matrices, as implied by the available diallel models. We overcame this gap by building the ‘model.matrixDiallel()’ function, which is tailored to build the correct model matrix for all the main equations in literature. Possible variations and enhancements (e.g. see Murray et al. 2003; Yao et al. 2013) can be accommodated within the same function.

Once the model matrix is defined, model fitting can be performed by usual tools, such as the ‘lm()’ function or the ‘lme.fit()’. For less experienced users, we built the ‘lm.diallel()’ function, that is a wrapper to the ‘lm.fit()’ function with a higher degree of usability, in relation to diallel models. The advantage of both approaches is that they can exploit several powerful methods for linear models, such as ‘summary()’, anova() or ‘glht()’ in the ‘multcomp’ package. In particular, the inspection of model residuals can be made in the very same fashion as for all other linear models, an aspect very frequently neglected in specialised diallel analyses softwares.

Increasing the usability of existing packages that have gained a wide popularity may be an advantageous programming strategy, compared to the usual strategy of building brand new platforms. From the point of view of the developer, it is efficient, as it requires a minor programming effort. From the point of view of the users (professionals, technicians and students), it is handy to be put in the conditions of making statistical analyses, without the need of learning new softwares and/or languages and/or syntaxes. In general, due to its open-source nature, the R environment is often overwhelming for users, that are confused by the extremely wide availability of alternative methods to perform the same task. In this regard, a programming strategy aimed at supporting some existing reference platforms might help build a more comfortable environment for statistical analyses.

One further aspect to be considered is that the dichotomy between random/mixed and fixed diallel models might be regarded as rather outdated; indeed, the availability of REML estimation has opened a third possibility, where we have a specific interest in the lines involved in the experiment, but, nonetheless, we model them as random. It is possible to obtain best linear unbiased predictors (BLUPs) that have been shown to be more accurate than best linear unbiased estimators (BLUEs) of genetical effects (Piepho et al. 2008). It is necessary to point out that good variance component estimates require a relatively high number of parents, which is not often the case with diallel experiments. Therefore, the use of OLS continues to be preferable for experiments conducted with a small number of parent lines, which motivated our initial focus on fixed effects model.

Nonetheless, our tools can also be useful to fit random effects and mixed effects diallel models. In particular, we have shown that it is possible to make use of the design matrices produced as the output of the ‘model.matrix()’ or ‘model.matrixDiallel()’ functions within the general purpose MCMC sampler JAGS; our fourth example relates to an experiment where the environment is random, while genetic effects are fixed, although a further extension to models with random genetic effects is immediate. Turner et al. (2018) showed that the Bayesian framework may be advantageous for fitting diallel models, while JAGS represents one of the most flexible and widespread general purpose MCMC samplers around. Both the framework and the sampler still deserve a wider appraisal in plant breeding. Obviously, such aspects as the selection of priors or the check for convergence are fundamental to sound analyses in the Bayesian framework and may require further attention and research by plant breeders. We did not consider these aspects as they appear to be beyond the scope of this manuscript and may require further work.

It is not clear whether and how our tools can be used for REML-based estimation of random diallel models. In principle, the design matrices as used in JAGS can also be used in other REML-based mixed model solvers, which are available within R packages, such as EMMREML (Akdemir and Godfrey 2015) and SAMM (Akdemir 2018), but further research is needed in this respect. For those who favour REML estimation, we provide a few examples on how random diallel models can be fitted in R by using the ‘sommer’ package (see Supplemental material).

## Electronic supplementary material

Below is the link to the electronic supplementary material.

Supplementary file 1 (DOCX 22 kb)

## Data Availability

All the above R functions are freely available in a gitHub repository (https://github.com/OnofriAndreaPG/lmDiallel).
