# Supplementary material for: Linear models for diallel crosses: a review with R functions
Source: Theor Appl Genet. 2020 Nov 6;134(2):585–601. doi: 10.1007/s00122-020-03716-8 (PMC7843492; doi:10.1007/s00122-020-03716-8)
Supplement: Supplementary file 1 — (DOCX 22 kb) [file 122_2020_3716_MOESM1_ESM.docx]

# Supplemental information

## *Bayesian analysis with JAGS*

The first step to a Bayesian analysis with JAGS is to specify an appropriate model (in JAGS code), which requires the following elements: 1. A ‘data’ step: the number of fixed and random parameters is derived, to be used in subsequent steps. 2. A ‘model’ step: priors are defined for all fixed and random terms. Random effects are defined by using a hyperprior parameter for the standard deviation. The likelihood of observations is defined by using Equation 11. Variance components are derived by squaring the standard deviations. Precisions are also derived by inverting the variances.

Box S1. JAGS definition of the model in Equation 11, for the ‘diallelMET’ dataset.

# Save BUGS description of the model to working directory
data {
n <- length(Y)
nf <- dim(X)
nb1 <- dim(Z.1)
nb2 <- dim(Z.2)
nb3 <- dim(Z.3)
nb4 <- dim(Z.4)
nb5 <- dim(Z.5)
nb6 <- dim(Z.6)
}

model {

# Definition of priors
beta[1] ~ dunif(0, 1000000)
for (i in 2:nf[2]){
beta[i] ~ dnorm(0, 0.0001)
}
for (i in 1:nb1[2]){
b1[i] ~ dnorm(0, tau.1)
}
for (i in 1:nb2[2]){
b2[i] ~ dnorm(0, tau.2)
}
for (i in 1:nb3[2]){
b3[i] ~ dnorm(0, tau.3)
}
for (i in 1:nb4[2]){
b4[i] ~ dnorm(0, tau.4)
}
for (i in 1:nb5[2]){
b5[i] ~ dnorm(0, tau.5)
}
for (i in 1:nb6[2]){
b6[i] ~ dnorm(0, tau.6)
}

sigma ~ dunif(0, 500)
sigma.1 ~ dunif(0, 500)
sigma.2 ~ dunif(0, 500)
sigma.3 ~ dunif(0, 500)
sigma.4 ~ dunif(0, 500)
sigma.5 ~ dunif(0, 500)
sigma.6 ~ dunif(0, 500)

# Likelihood
for (i in 1:n) {
 expected[i] <- inprod(X[i,], beta) + inprod(Z.1[i,], b1) + inprod(Z.2[i,], b2) + inprod(Z.3[i,], b3) + inprod(Z.4[i,], b4) + inprod(Z.5[i,], b5) + inprod(Z.6[i,], b6)
 Y[i] ~ dnorm(expected[i], tau)
}

# Derived quantities
sigma2 <- sigma * sigma
sigma2.1 <- sigma.1 * sigma.1
sigma2.2 <- sigma.2 * sigma.2
sigma2.3 <- sigma.3 * sigma.3
sigma2.4 <- sigma.4 * sigma.4
sigma2.5 <- sigma.5 * sigma.5
sigma2.6 <- sigma.6 * sigma.6
tau <- 1 / sigma2
tau.1 <- 1 / sigma2.1
tau.2 <- 1 / sigma2.2
tau.3 <- 1 / sigma2.3
tau.4 <- 1 / sigma2.4
tau.5 <- 1 / sigma2.5
tau.6 <- 1 / sigma2.6
}

## *REML-based analyses with ‘sommer’*

For those who prefer REML-based analyses with respect to bayesian analyses, we report the R code to reproduce the results obtained in Möhring and Piepho (2011), by using the ‘sommer’ package. In this case, all genetic effects are random and the analyses can be perfomed by an accurate definition of ‘dummy’ variables, while our matrix facilities are not needed. In more detail, with the following code we reproduce the results of the first example in Möhring and Piepho (2011), which uses the same flowering date data as in our first example. Variance components estimates of genetic effects are shown in Table 2 of the forementioned paper and small differences in our results are only numerical, while model specifications are equivalent.

Box S2. Estimating variance components for genetic effects by REML method, as shown in Example 1 of Möhring and Piepho (2011).

# Reproducing the results in Table 2 of Möhring and Piepho (2011)
rm(list = ls())
library(sommer)
library(lmDiallel)

data("hayman54")
df <- hayman54

# Dummy variables for selfs, crosses, combinations
df$crosses <- ifelse(df$Par1 == df$Par2, 0, 1)
df$selfs <- ifelse(df$Par1 == df$Par2, 1, 0)
df$dr <- ifelse(as.character(df$Par1) < as.character(df$Par2), -1,
 ifelse(as.character(df$Par1) == as.character(df$Par2), 0, 1))

# Dummy variable for the combinations, ignoring the reciprocals
df$combination<-factor(ifelse(as.character(df$Par1) <= as.character(df$Par2),
 paste(df$Par1, df$Par2, sep =""),
 paste(df$Par2, df$Par1, sep ="")) )


# GRIFFING MODEL 2 with reciprocal effects ################################
mod1h <- mmer(Ftime ~ 1, data=df,
 random = ~ Block
 + overlay(Par1, Par2)
 + combination
 + combination:dr)

summary(mod1h)$varcomp

## VarComp VarCompSE Zratio
## Block.Ftime-Ftime 0.00000 9.32181 0.000000
## overlay(Par1, Par2).Ftime-Ftime 1276.73089 750.17269 1.701916
## combination.Ftime-Ftime 1110.99090 330.16921 3.364914
## combination:dr.Ftime-Ftime 66.02295 49.26876 1.340057
## units.Ftime-Ftime 418.47949 74.56442 5.612321

# GE2 model, no reciprocal effects #######################################
mod1h <- mmer(Ftime ~ Block + crosses, data=df,
 random = ~ overlay(Par1, Par2) #VEi
 + overlay(Par1, Par2):crosses #h.i
 + combination:crosses) # SCA

summary(mod1h)$varcomp

## VarComp VarCompSE Zratio
## overlay(Par1, Par2).Ftime-Ftime 2304.1781 1261.63193 1.826347
## overlay(Par1, Par2):crosses.Ftime-Ftime 613.6040 402.74347 1.523560
## combination:crosses.Ftime-Ftime 340.7030 148.56225 2.293335
## units.Ftime-Ftime 501.6275 74.36075 6.745864

# GE3, no reciprocal effects ############################################
mod1h <- mmer(Ftime ~ Block + crosses, data=df,
 random = ~ overlay(Par1, Par2):crosses # GCAC
 + Par1:selfs # SP
 + combination:crosses) # SCA

summary(mod1h)$varcomp

## VarComp VarCompSE Zratio
## overlay(Par1, Par2):crosses.Ftime-Ftime 927.7895 537.91218 1.724797
## Par1:selfs.Ftime-Ftime 9960.9247 5456.58188 1.825488
## combination:crosses.Ftime-Ftime 341.4567 148.53667 2.298804
## units.Ftime-Ftime 498.5974 73.92066 6.745035

# GE3 with reciprocal effects ###########################################
# In Möhring: mixed model 3 reduced
mod1h <- mmer(Ftime ~ Block + crosses, data=df,
 random = ~ overlay(Par1, Par2):crosses #GCAC
 + Par1:selfs # SP
 + combination:crosses # SCA
 + combination:dr) # REC

summary(mod1h)$varcomp

## VarComp VarCompSE Zratio
## overlay(Par1, Par2):crosses.Ftime-Ftime 927.78742 537.89981 1.724833
## Par1:selfs.Ftime-Ftime 10001.78854 5456.47578 1.833013
## combination:crosses.Ftime-Ftime 361.89712 148.54264 2.436318
## combination:dr.Ftime-Ftime 66.43695 49.24492 1.349113
## units.Ftime-Ftime 416.82960 74.27202 5.612203

# GE3 with RGCA + RSCA ##################################################
# In Möhring: mixed model 3
mod1h <- mmer(Ftime ~ Block + crosses, data=df,
 random = ~ overlay(Par1, Par2):crosses #GCAC
 + Par1:selfs # SP
 + combination:crosses # SCA
 + overlay(Par1,Par2):dr # RGCA: exclude selfs
 + combination:dr) #RSCA: exclude selfs

summary(mod1h)$varcomp

## VarComp VarCompSE Zratio
## overlay(Par1, Par2):crosses.Ftime-Ftime 927.7843 537.88164 1.7248857
## Par1:selfs.Ftime-Ftime 10001.7570 5456.30125 1.8330654
## combination:crosses.Ftime-Ftime 361.8958 148.53670 2.4364068
## overlay(Par1, Par2):dr.Ftime-Ftime 17.9799 19.92428 0.9024114
## combination:dr.Ftime-Ftime 30.9519 46.43908 0.6665054
## units.Ftime-Ftime 416.8274 74.26813 5.6124668
